# Gut microbiota, immune cell traits, and infectious mononucleosis: a two-sample mendelian randomization and mediation analysis

**DOI:** 10.1590/1414-431X2026e15657

**Published:** 2026-11-09

**Authors:** Guolian Wu, Meiling Wang, Yonghua Si, Xue Wang, Hongna Li, Lili Wang, Rong Wang

**Affiliations:** 1 Department of Pediatrics, Cangzhou People's Hospital, Cangzhou, Hebei Province, China

**Keywords:** Gut microbiota, Infectious mononucleosis, Immune-cell traits, Mendelian randomization, Mediation analysis

## Abstract

Infectious mononucleosis (IM) is an acute disease caused by the Epstein-Barr virus. This study assessed the causal association between the gut microbiota (GM) and IM and how the immune cells are mediators of this process through Mendelian randomization (MR). Two-step, two-sample MR was used to test the overall causal impact of GM on IM, the impact of GM on 731 immune cell characteristics, and the indirect effect of GM on IM. GM summary statistics (207 taxa, 7,738 Dutch participants), immune cell genome-wide association studies (GWAS, 3,757 Sardinians), and IM data (3,838 cases/486,087 controls, FinnGen R12) were used. P<1e−5, LD r^2^<0.001, and F>10 were used as the criteria in selecting instrumental variables. Bidirectionality MR and mediation MR with 1000 bootstrap replications were done to determine reverse causality and proportional mediation effects. MR analysis revealed 12 GM taxa with protective effects against IM and three taxa associated with an increased risk of IM. *G__Lachnospiraceae_noname* increased IM, and *o__Burkholderiales.f__Oxalobacteraceae* and *g__Oxalobacter_formigenes* had protective effects. IM had no impact on GM composition, and 22 immune cell characteristics were causally associated with IM with 10 risk-enhancing and 12 protective traits. The increased absolute count of hematopoietic stem cells (HSC AC) mediated 10.4% and the reduced percentage of CM CD8br %CD8br mediated -13.3% of the protective effect of *o__Burkholderiales.f__Oxalobacteraceae*. The results offer an important piece of information on microbial and immunologic targets of IM prevention.

## Introduction

Infectious mononucleosis (IM) is a viral infection mostly affecting adolescents and young adults that is triggered by Epstein-Barr virus (EBV), an ubiquitous virus that belongs to the γ-herpesvirus family (1). In addition to IM, EBV is implicated in a range of malignancies and autoimmune diseases, which further increases the clinical relevance of this virus (2). IM clinically manifests with typical symptoms of fever, pharyngitis, lymphadenopathy, and deep fatigue, regularly lasting several weeks to months (3). The patient can also have serious complications, such as splenic rupture, fulminant hepatitis, and neurological sequelae, which may result in death (4). The available treatment of IM is primarily based on symptomatic and supportive treatment as the antiviral medications and immunomodulatory therapies are not that useful in clinical practice. The absence of effective specific treatment and the long-term effects of IM indicate an urgent need to unravel its pathogenic mechanisms. The purpose of the study was to determine the main causal elements of IM susceptibility, which will help develop new intervention strategies and enhance clinical management.

The gut microbiota (GM) has recently emerged as an important focus in studies of immune regulation and susceptibility to infectious diseases. A large body of evidence shows that the GM controls the host immune system, influencing the pathogenesis of a number of infectious and autoimmune diseases, the control of inflammatory response, antigen recognition, and immune cell differentiation and functions (5,6). It is worth mentioning that GM dysbiosis is directly associated with impaired immune homeostasis and the formation of an inflammatory microenvironment, which directly influences the immune susceptibility to EBV infection and IM progression (7). Immune cells are also fundamental elements of pathogen defense in the body and are also crucial in the development of IM (8). Nevertheless, most existing research is limited to examining correlations between the GM and specific immune phenotypes; systematic studies of causal association between GM, immune cells, and IM pathogenesis are still lacking.

The present study used a Mendelian randomization (MR) approach, in which genetic variants serve as instrumental variables, to systematically investigate the causal relationships among the GM, immune cell characteristics, and IM. MR takes advantage of the laws of inheritance proposed by Mendel by treating randomly assigned genetic variants as a natural experiment, thereby minimizing confounding bias and reverse causation, which commonly limit conventional observational studies (9). Over the past few years, MR has been widely used in epidemiology, GM research, and immunology and has been demonstrated to be a highly reliable method for causal inference (10). Moreover, bidirectional MR analyses were conducted to eliminate potential reverse causation among these three components, thereby providing robust genetic evidence to clarify the etiological mechanisms underlying IM.

The present study used large-scale genetic epidemiological data and MR approaches to comprehensively examine the causal relationships between GM and immune cell characteristics, as well as the causal effects of both GM and immune cell characteristics on IM risk. By combining multiple genetic instrumental variables with complementary statistical methods for cross-validation, this study aimed to identify the major pathways of the gut-immune axis involved in IM pathogenesis while minimizing the influence of confounding factors and reverse causation. This work not only enhanced our understanding of IM etiology but also provided a theoretical foundation and identified potential targets for the precise prevention and personalized treatment of IM.

## Material and Methods

### Study design

To explore the causal associations among GM, immune cell characteristics, and IM, we performed a two-sample and mediation MR study, as shown in Figure 1. For the MR analysis to be valid, the instrumental variables (IVs) had to satisfy three core assumptions: i) the selected genetic variants were strongly associated with GM or immune cell traits (relevance assumption); ii) the genetic variants were independent of potential confounding factors (independence assumption); iii) the genetic variants influenced IM only through the exposure of interest (GM or immune cell traits), rather than through horizontal pleiotropy (exclusion restriction assumption).

**Figure 1:**
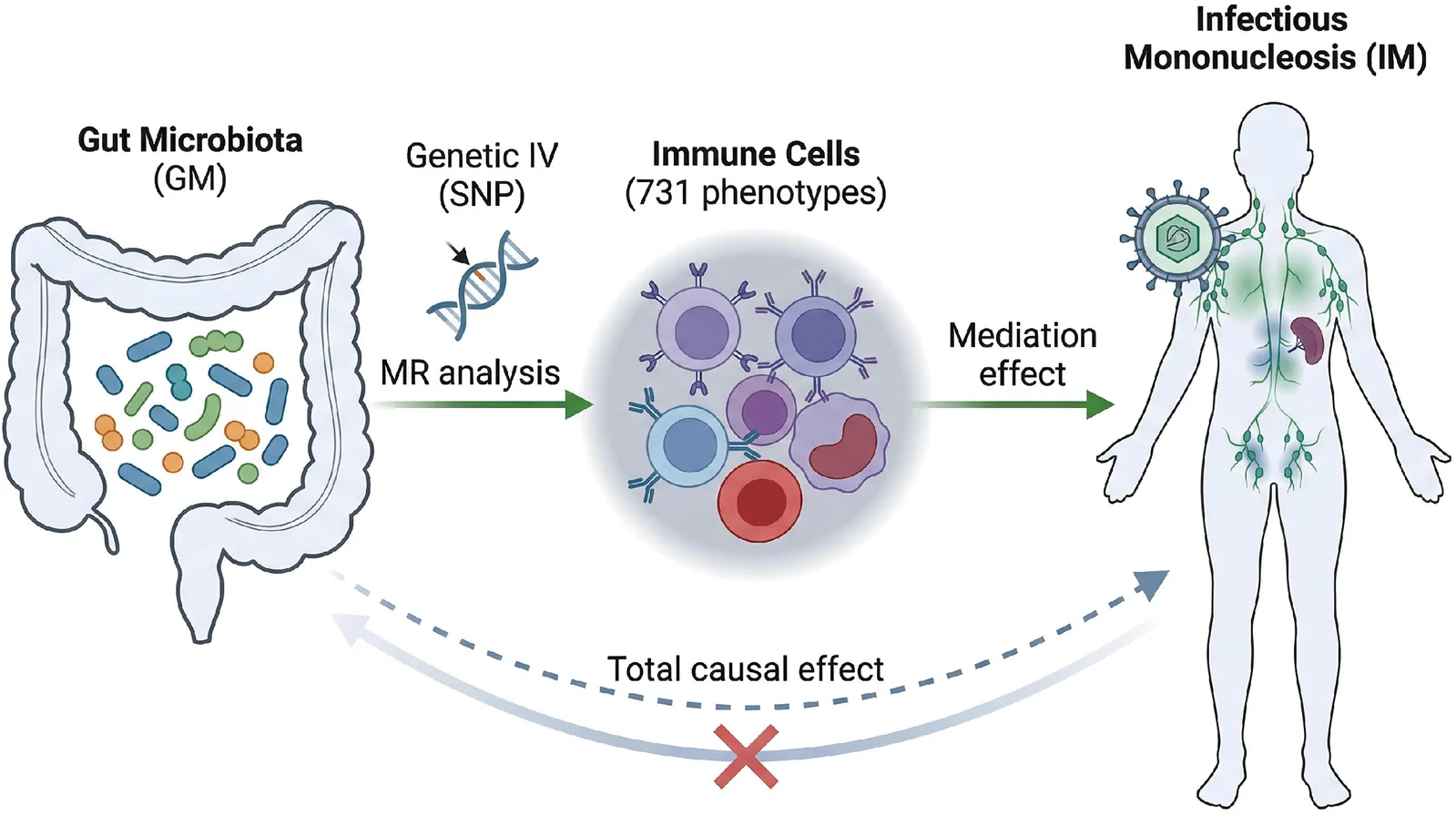
Flow chart of the study. The flowchart was generated using Jova AI (Jie Luoxuan AI, http://jova.ai). All key nodes and regulatory pathways were verified by the authors. IV: instrumental variables; SNP: single nucleotide polymorphism; MR: Mendelian randomization.

### Data sources

This paper utilized publicly released, ethics-approved GWAS summary data (anonymized, de-identified) that had no direct human interactions or primary sample. It was carried out according to the Declaration of Helsinki, therefore no specification was required regarding any ethics.

The GM summary statistics were obtained from a Dutch microbial genome-wide association study (GWAS) that included metagenomic sequencing data from 7,738 participants in the northern Netherlands (11). This GWAS investigated the relationship between host genetics and the GM by testing associations with 207 microbial taxa and 205 functional pathways. Immune cell trait genomic data were obtained from a GWAS of 3,757 Sardinian individuals, including 731 immune phenotypes (GCST0001391 to GCST0002121) (12). This dataset comprised 539 immune cell phenotypes and 192 relative cell count traits, including morphological characteristics, median fluorescence intensities of surface antigens, and cell counts. These immunophenotypes provide insights into the complex genetic regulation of immune cell subtypes in autoimmune diseases. IM genetic data were retrieved from the FinnGen database (finngen_R12_AB1_EBV.gz), which included 3,838 IM cases and 486,087 controls.

### Selection of instrumental variables

To select IVs associated with GM, we used a significance threshold of P<1e−5, which is consistent with previous GM studies (13). We also ensured that the candidate single nucleotide polymorphims (SNPs) were independent by applying a linkage disequilibrium threshold of r^2^ <0.001 and a physical distance threshold of 10,000 kb (14). Moreover, only SNPs with an F-statistic >10 were retained to minimize weak instrument bias and ensure a strong genetic association with the exposure (GM or immune cell traits) (15).

### Statistical analysis

R software (version 4.3.3) was used for all statistical analyses, and two-sample MR analyses were performed using the TwoSampleMR, VariantAnnotation, and ieugwasr packages (16). Five MR methods were used: MR-Egger, weighted median, inverse variance weighted (IVW), simple mode, and weighted mode. Among these, the IVW method was selected as the primary analytical approach because of its high statistical power and accuracy in summary-data MR when the underlying assumptions are satisfied. A two-tailed P<0.05 was considered indicative of a causal relationship between the exposure and the outcome. To account for multiple comparisons in the large-scale omics analysis, the false discovery rate (FDR) was used to control for multiple testing while maintaining statistical power (17). Associations with an FDR <0.05 were considered statistically significant. Heterogeneity was assessed using Cochran's Q statistic under both the IVW and MR-Egger methods. Horizontal pleiotropy was evaluated using the MR-Egger intercept test and the MR-PRESSO (Mendelian Randomization Pleiotropy RESidual Sum and Outlier) method. MR-PRESSO was also used to compare the causal estimates before and after outlier SNP removal to assess the influence of outliers (16). Leave-one-out analysis was conducted to evaluate the influence of each candidate SNP on the overall causal estimate, further supporting the robustness of the results (18).

We investigated the mediating role of immune cell traits in the causal relationship between GM and IM using a two-step MR method. First, univariate MR analysis was performed to estimate the total causal effect of GM on IM (β_total). Secondly, the two-sample MR was conducted to estimate the causal effect of GM on the intermediate immune cell traits (β_1_). Third, the causal effect of immune cell traits on IM (β_2_) was estimated using multivariate MR, adjusting for GM to account for its direct effect. The indirect causal effect of GM on IM mediated through immune cell characteristics was calculated as β_1_×β_2_, whereas the direct causal effect of GM on IM (not mediated by immune cell characteristics) was calculated as the difference between the total and indirect effects (β_direct = β_total − β_indirect). The percentage of mediation was calculated as (indirect effect / total effect) × 100% (19).

To improve the precision of the mediation estimates and provide appropriate statistical power for estimating confidence intervals (CI), we used the bootstrap method with 1,000 replications. Bias-corrected and accelerated (BCa) method was used to obtain 95%Cis, and P values were derived from the bootstrap sampling distribution (20). All mediation analyses were performed in R using the boot package.

## Results

### Effect of GM on IM

Based on the stringent IV selection criteria, we identified three GM taxa that showed significant causal relationship with IM. Figure 2 and Table 1 present the causal effects of each GM taxon on IM. Among the three taxa associated with IM, *g__Lachnospiraceae_noname.s__Lachnospiraceae_bacterium_8_1_57FAA* was significantly associated with an increased risk of IM (OR=1.093, 95%CI: 1.018-1.173, P=0.014, q=0.042). Conversely, *o__Burkholderiales.f__Oxalobacteraceae* (OR=0.873, 95%CI: 0.767-0.993, P=0.038, q=0.057) and *g__Oxalobacter.s__Oxalobacter_formigenes* (OR=0.873, 95%CI: 0.767-0.993, P=0.038, q=0.038) were associated with a protective effect against IM.

**Figure 2:**
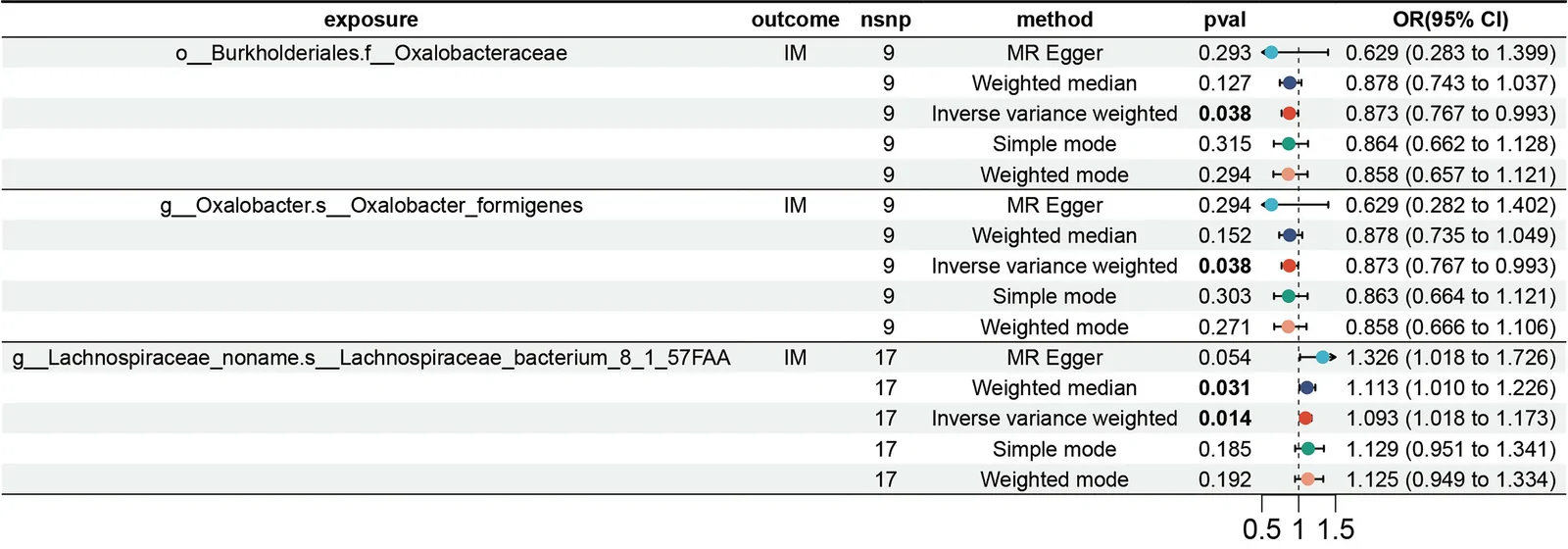
Mendelian randomization (MR) analysis showed the associations between gut microbiota taxa and infectious mononucleosis (IM). nsnp: number of single nucleotide polymorphisms; OR: odds ratio; CI: confidence interval.

**Table 1:** Mendelian randomization (MR) estimates of the causal relationships between 3 gut microbiota taxa and risk of infectious mononucleosis.

Exposure	Method	nSNP	β	SE	P-value	OR	95%CI		q-value
							lower	upper	
*g__Lachnospiraceae_noname.s__Lachnospiraceae_bacterium_8_1_57FAA*	MR Egger	17	0.282	0.135	0.054	1.326	1.018	1.726	
	Weighted median	17	0.107	0.050	0.031	1.113	1.010	1.226	
	IVW	17	0.089	0.036	0.014	1.093	1.018	1.173	0.042
	Simple mode	17	0.122	0.088	0.185	1.129	0.951	1.341	
	Weighted mode	17	0.118	0.087	0.192	1.125	0.949	1.334	
*o__Burkholderiales.f__Oxalobacteraceae*	MR Egger	9	-0.463	0.408	0.293	0.629	0.283	1.399	
	Weighted median	9	-0.130	0.085	0.127	0.878	0.743	1.037	
	IVW	9	-0.136	0.066	0.038	0.873	0.767	0.993	0.057
	Simple mode	9	-0.146	0.136	0.315	0.864	0.662	1.128	
	Weighted mode	9	-0.153	0.136	0.294	0.858	0.657	1.121	
*g__Oxalobacter.s__Oxalobacter_formigenes*	MR Egger	9	-0.463	0.409	0.294	0.629	0.282	1.402	
	Weighted median	9	-0.130	0.084	0.124	0.878	0.744	1.036	
	IVW	9	-0.136	0.066	0.038	0.873	0.767	0.993	0.038
	Simple mode	9	-0.147	0.139	0.320	0.863	0.658	1.133	
	Weighted mode	9	-0.153	0.122	0.244	0.858	0.676	1.089	

nSNP: number of single nucleotide polymorphisms; IVW: inverse variance weighted; OR: odds ratio; CI: confidence interval.

In this MR analysis, Cochran's Q statistic, the MR-Egger intercept test, and MR-PRESSO indicated no evidence of heterogeneity or horizontal pleiotropy (Supplementary Table S1). Furthermore, leave-one-out sensitivity analysis showed that no individual SNP disproportionately influenced the overall causal effect of GM on IM (Figure 3).

**Figure 3:**
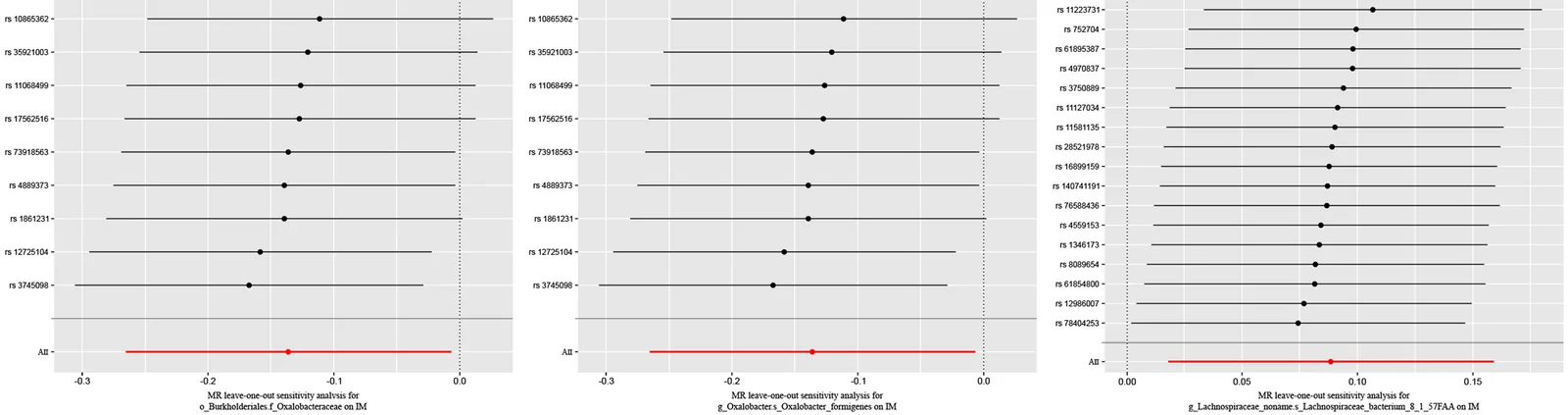
Leave-one-out sensitivity analysis of the causal effect of gut microbiota on infectious mononucleosis (IM). **A**, *o__Burkholderiales.f__Oxalobacteraceae.* **B**, *g_Oxalobacter.s_Oxalobacter_formigenes.* **C**, g*_Lachnospiraceae_noname.s_Lachnospiraceae_bacterium_8_1_57FAA.*

### Effect of immune cell traits on IM

Using the stringent IV selection criteria, we identified 22 immune cell traits that showed significant causal relationship with IM. The causal effects of these traits are presented in Supplementary Figure S1 and Table 2. Among these immune cell traits, 10 were positively associated with an increased risk of IM, whereas 12 were negatively associated with a reduced risk of IM. Notably, the expression of CD62L on CD62L^+^ plasmacytoid DCs showed the strongest risk-promoting effect (OR = 1.150, 95%CI: 1.056-1.251, P=0.001, q=0.009). In contrast, IgD^−^ CD38dim exhibited the strongest protective effect (OR=0.898, 95%CI: 0.825-0.977, P=0.012, q=0.044).

**Table 2:** MR: Mendelian randomization (MR) estimates of the causal relationships between 22 immune cell traits and risk of infectious mononucleosis.

Exposure	Method	nSNP	P-value	OR	OR (95%CI)		q	Pleiotropy	Heterogeneity		
					lower	upper		P-value	Q-value	Q-df	P-value
BAFF-R on IgD^−^ CD38br	IVW	15	0.019	0.910	0.841	0.985	0.048	0.677	10.467	14	0.727
CD127 on granulocyte	IVW	30	0.032	1.068	1.006	1.135	0.050	0.576	21.177	29	0.853
CD16-CD56 on HLA DR+ NK	IVW	20	0.044	0.921	0.850	0.998	0.048	0.657	12.999	19	0.839
CD25 on CD45RA- CD4 not Treg	IVW	19	0.027	1.128	1.014	1.255	0.049	0.592	26.801	18	0.083
CD25 on CD39+ CD4+	IVW	17	0.006	0.930	0.883	0.979	0.026	0.587	8.678	16	0.926
CD28 on CD28+ CD45RA+ CD8br	IVW	13	0.017	0.908	0.838	0.983	0.053	0.935	13.906	12	0.307
CD28 on CD39+ secreting Treg	IVW	16	0.017	1.104	1.018	1.197	0.054	0.460	18.429	15	0.241
CD28 on CD45RA- CD4 not Treg	IVW	13	0.005	0.903	0.841	0.969	0.026	0.926	11.957	12	0.449
CD28+ CD45RA+ CD8dim %CD8dim	IVW	19	0.001	1.129	1.050	1.215	0.012	0.135	10.955	18	0.896
CD3 on HLA DR+ T cell	IVW	23	0.031	1.070	1.006	1.138	0.052	0.294	16.464	22	0.792
CD33br HLA DR+ CD14dim %CD33br HLA DR+	IVW	20	0.037	0.931	0.870	0.996	0.051	0.674	21.742	19	0.297
CD39+ CD8br %T cell	IVW	19	0.041	0.918	0.846	0.996	0.050	0.456	23.548	18	0.170
CD4 on activated Treg	IVW	19	0.035	1.096	1.007	1.193	0.051	0.555	20.606	18	0.300
CD45RA- CD28- CD8br %CD8br	IVW	19	0.001	1.009	1.004	1.015	0.019	0.693	16.055	18	0.589
CD62L on CD62L+ plasmacytoid DC	IVW	13	0.001	1.150	1.056	1.251	0.009	0.485	9.578	12	0.653
CM CD8br %CD8br	IVW	29	0.020	0.917	0.852	0.986	0.043	0.570	16.297	28	0.961
FSC-A on NKT	IVW	18	0.049	0.904	0.818	0.999	0.049	0.496	14.698	17	0.617
Granulocyte %leukocyte	IVW	21	0.024	0.904	0.829	0.987	0.048	0.405	14.365	20	0.811
HLA DR+ CD4+ AC	IVW	20	0.043	1.071	1.002	1.145	0.049	0.641	14.915	19	0.728
HSC AC	IVW	15	0.046	0.928	0.863	0.999	0.049	0.540	8.242	14	0.876
IgD^−^ CD38dim %B cell	IVW	19	0.012	0.898	0.825	0.977	0.044	0.812	20.361	18	0.313
TD CD8br AC	IVW	17	0.039	1.112	1.006	1.230	0.050	0.893	11.312	16	0.790

IVW: inverse variance weighted; nSNP: number of single nucleotide polymorphisms.

Sensitivity analyses showed no evidence of horizontal pleiotropy or heterogeneity for any of the 22 immune cell traits, as indicated by non-significant MR-Egger intercept and Cochran's Q tests (all P>0.05), supporting the validity of the causal estimates.

### Effect of GM on immune cell traits

Having identified three GM taxa and 22 immune cell traits that were causally associated with IM, we next examined the causal relationships between these 3 GM taxa and the 22 immune cell traits using MR analysis. Among the 3 GM taxa, only *o__Burkholderiales.f__Oxalobacteraceae* showed significant associations, specifically with CM CD8br %CD8br (OR=0.816, 95%CI: 0.688-0.967, P=0.019), and hematopoietic stem cell absolute count (HSC AC) (OR=1.209, 95%CI: 1.019-1.434, P=0.030). Consistent with these findings, the weighted median analysis confirmed significant associations between *o__Burkholderiales.f__Oxalobacteraceae* and CM CD8br %CD8br (OR=0.791, 95%CI: 0.650-0.961, P=0.019). No significant causal associations were detected using the other MR methods (MR-Egger, simple mode, or weighted mode) (Figure 4 and Table 3). Moreover, no evidence of heterogeneity or horizontal pleiotropy was detected, and leave-one-out analysis indicated that no single SNP disproportionately influenced the causal estimates (Supplementary Table S2).

**Figure 4:**
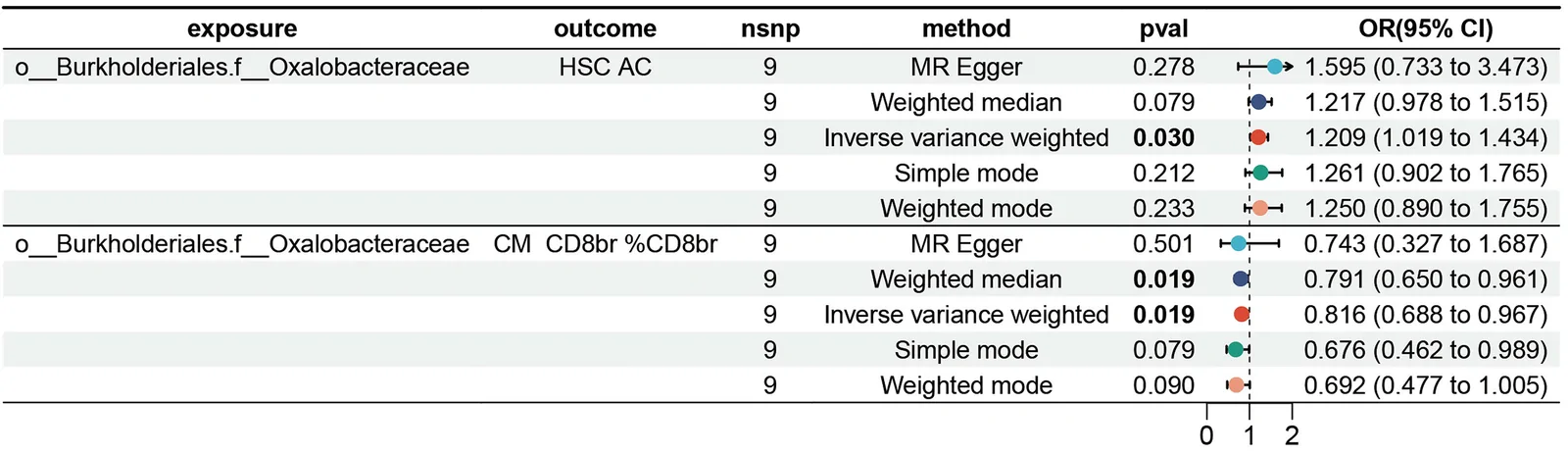
Forest plots demonstrating the causal associations between the gut microbiota and immune cell traits. HSC AC: Hematopoietic stem cell absolute count; nsnp: number of single nucleotide polymorphisms; MR: Mendelian randomization; OR: odds ratio; CI: confidence interval.

**Table 3:** Mendelian randomization (MR) analysis between gut microbiota and mediator.

Exposure	Outcome	Method	nSNP	β	SE	P-value	OR	OR (95%CI)		q-value
								lower	upper	
*o__Burkholderiales.f__Oxalobacteraceae*	CM CD8br %CD8br	MR Egger	9	-0.297	0.418	0.501	0.743	0.327	1.687	0.038
		Weighted median	9	-0.235	0.100	0.019	0.791	0.650	0.961	
		IVW	9	-0.204	0.087	0.019	0.816	0.688	0.967	
		Simple mode	9	-0.392	0.194	0.079	0.676	0.462	0.989	
		Weighted mode	9	-0.368	0.190	0.090	0.692	0.477	1.005	
	HSC AC	MR Egger	9	0.467	0.397	0.278	1.595	0.733	3.473	0.030
		Weighted median	9	0.197	0.112	0.079	1.217	0.978	1.515	
		IVW	9	0.189	0.087	0.030	1.209	1.019	1.434	
		Simple mode	9	0.232	0.171	0.212	1.261	0.902	1.765	
		Weighted mode	9	0.223	0.173	0.233	1.250	0.890	1.755	

HSC AC: hematopoietic stem cell absolute count; IVW: inverse variance weighted; nSNP: number of single nucleotide polymorphisms; OR: odds ratio; CI: confidence interval.

### Reverse MR analysis

We identified causal associations between three GM taxa and IM, as well as between 22 immune cell traits and IM. In addition, reverse MR analyses of the GM-IM and immune cell trait-IM relationships showed no significant reverse causal effects, supporting the directionality of the observed associations (Supplementary Table S3).

### Mediation analysis result

To further elucidate the causal relationship between *o__Burkholderiales.f__Oxalobacteraceae* and IM, we performed a mediation analysis to evaluate whether HSC AC and CM CD8br %CD8br mediated this association (Figure 5). The mediation analysis showed that HSC AC had an indirect effect of −0.014 (95%CI: −0.015-0.014; P<1e−10), accounting for 10.4% of the total effect. In contrast, CM CD8br %CD8br had an indirect effect of 0.018 (95%CI: 0.018-0.019; P<1e−10), corresponding to a mediation proportion of −13.3% of the overall effect (Table 4).

**Figure 5:**
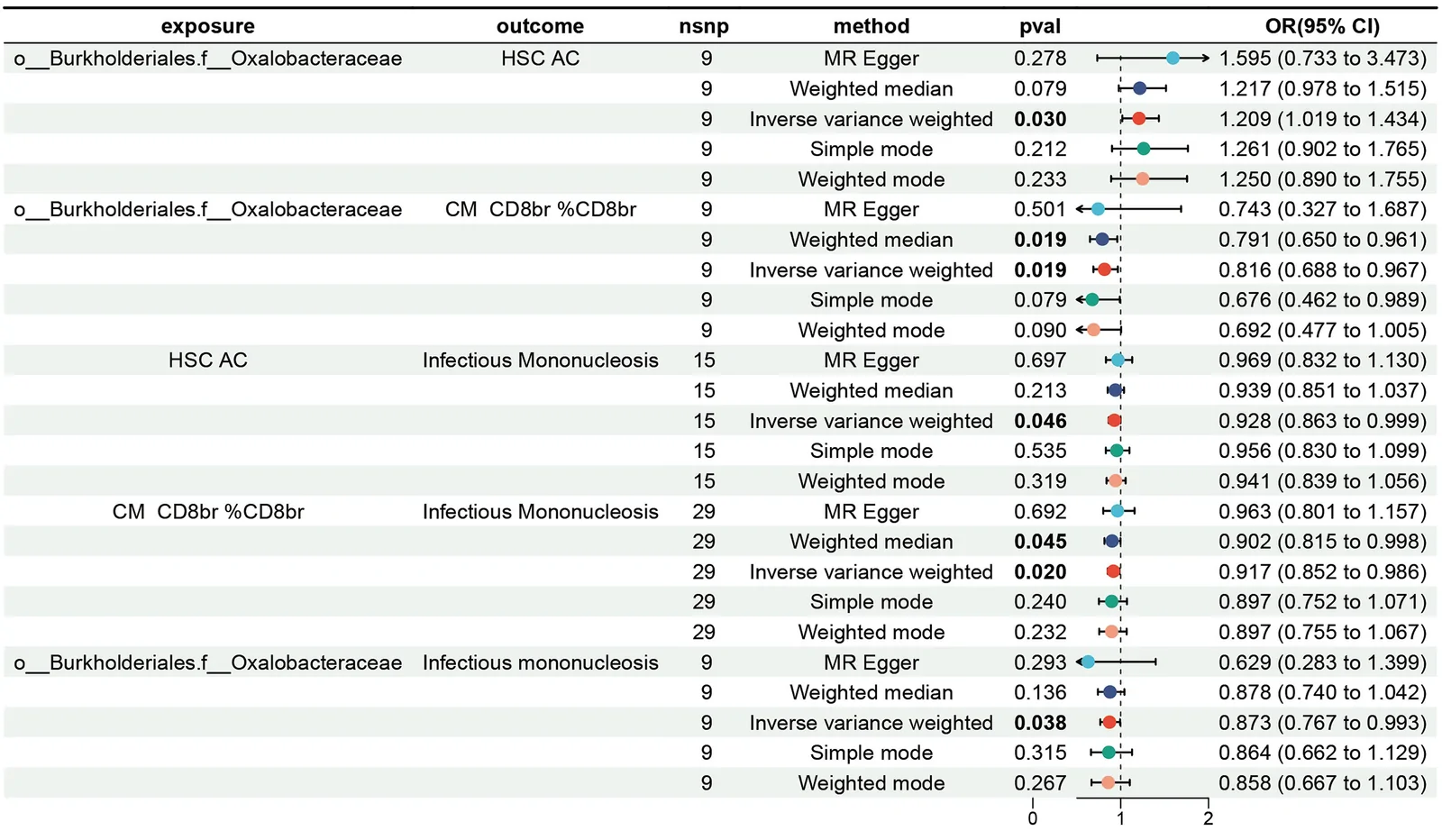
Forest plot shows the Mendelian randomization (MR) results of gut microbiota on infectious mononucleosis mediated by immune cells. HSC AC: hematopoietic stem cell absolute count; nsnp: number of single nucleotide polymorphisms; OR: odds ratio; CI: confidence interval.

**Table 4:** Mediation effect of gut microbiota on infectious mononucleosis via immune cells.

Exposure	Mediator	β all	β1	β2	Mediated effect(95%CI)	Direct effect	Mediated proportion (%)	P-value
*o__Burkholderiales.f__Oxalobacteraceae*	CM CD8br %CD8br	-0.136	-0.204	-0.087	0.018 (0.018, 0.019)	-0.154	-13.3%	<1e-10
	HSC AC	-0.136	0.189	-0.074	-0.014 (-0.015, -0.014)	-0.122	10.4%	<1e-10

HSC AC: hematopoietic stem cell absolute count.

## Discussion

This study used a two-sample MR design to comprehensively investigate the causal associations and mediating roles of GM and immune cell traits in IM using large-scale GWAS data. The main results indicated a multidimensional regulatory network in which three GM taxa were directly associated with IM susceptibility, 22 immune cell traits independently influenced IM risk, and *o__Burkholderiales.f__Oxalobacteraceae* exhibited an indirect association with IM through two immune cell mediators (CM CD8br %CD8br and HSC AC). These findings provided new insights into the etiological mechanisms of IM and identified potential targets for disease prevention and intervention.

Three GM taxa showed significant causal associations with IM. *g__Lachnospiraceae_noname.s__Lachnospiraceae_bacterium_8_1_57FAA* was associated with an increased risk of IM, whereas *o__Burkholderiales.f__Oxalobacteraceae* and *g__Oxalobacter.s__Oxalobacter_formigenes* showed consistent protective effects. These findings are consistent with the established role of GM dysbiosis in modulating susceptibility to viral infections through the regulation of immune responses (21). As a major family of gut commensals, Lachnospiraceae is generally associated with short-chain fatty acid (SCFA) production and maintenance of intestinal barrier integrity (22). Nevertheless, to our knowledge, this is the first study to provide genetic evidence that a specific unnamed taxon within the Lachnospiraceae family may increase susceptibility to IM, possibly by disrupting mucosal immune homeostasis and facilitating EBV infection. This finding is consistent with previous cross-sectional evidence showing increased Lachnospiraceae abundance in EBV-seropositive individuals (23). The protective effects of *o__Burkholderiales.f__Oxalobacteraceae* and *g__Oxalobacter.s__Oxalobacter_formigenes* are biologically plausible, as *Oxalobacter formigenes* has been reported to modulate immune responses against viral infections (24,25). The identical ORs and highly similar confidence intervals observed for these two taxa may reflect functional redundancy or synergistic protective effects within the gut microbial community, providing additional insights into host-microbiome interaction.

Twenty-two immune cell traits were significantly associated with IM, including 10 risk-enhancing and 12 protective traits. CD62L expression on CD62L^+^ plasmacytoid DCs (pDCs) showed the strongest risk-promoting effect, while IgD^−^ CD38dim %B cells exhibited the strongest protective effect. These findings highlight the central role of subtype-specific immune responses in regulating EBV infection and provide genetic evidence supporting causal relationships suggested by previous immunological studies. As the major producers of type I interferons (IFN-α/β), pDCs rely on CD62L-mediated homing to secondary lymphoid tissues to initiate antiviral immune responses (26). The positive association between elevated CD62L expression and increased IM risk suggests that excessive activation of pDCs to lymphoid tissues might result in immune dysregulation, whereas impaired migration of pDCs to infection sites may reduce antiviral activity. This interpretation is consistent with experimental data showing that mice lacking pDC-derived IFN-α exhibit accelerated EBV replication (27,28). The protective effect of IgD^−^ CD38dim %B cells also provides genetic support for the clinical hypothesis that EBV-specific memory B cells contribute to protection against recurrent IM (29). Moreover, to our knowledge, this is the first study to genetically identify additional immune cell subsets, including CD25^+^ T cells and CD16^−^CD56^+^ NK cells, as being associated with IM, highlighting the potential roles of T-cell activation and NK-cell cytotoxicity in IM pathogenesis (30).

One of the major novel findings of this study is that *o__Burkholderiales.f__Oxalobacteraceae* may indirectly influence IM through two immune cell subtypes. Specifically, it was negatively associated with CM CD8br %CD8br and positively associated with HSC AC, with mediation proportions of −13.3% and 10.4%, respectively. These findings provide genetic evidence supporting the involvement of the gut-immune axis in IM pathogenesis, consistent with experimental studies showing that gut-derived short-chain fatty acids (SCFAs) regulate both CD8^+^ T-cell memory formation and HSC proliferation (31,32). The negative mediation effect of CM CD8br %CD8br suggests that *o__Burkholderiales.f__Oxalobacteraceae* may promote the differentiation of central memory CD8^+^ T cells into effector cells, thereby enhancing the immediate antiviral response (33). In contrast, the positive mediation effect of HSC AC may reflect enhanced HSC activity, facilitating the *de novo* production of antiviral T cells and promoting immune recovery following infection (34). Together, these findings support a complex bidirectional gut microbiota-immune cell regulatory network rather than a simple unidirectional GM-immune cell pathway, providing new insights into the multistep mechanisms underlying IM.

Extensive sensitivity analyses, including Cochran's Q test, the MR-Egger intercept test, MR-PRESSO, and leave-one-out analysis, together with reverse MR analyses, showed no evidence of significant heterogeneity, horizontal pleiotropy, or reverse causality in the identified causal relationships. These findings support the robustness of the causal inferences obtained using stringent instrumental variable selection criteria (P<1e−5 r^2^<0.001, F-statistic>10).

Nonetheless, this study has several limitations. First, the GWAS data for GM and immune cell traits were derived from populations of European ancestry (Dutch and Sardinian), which may limit the generalizability of the findings to other non-European ethnicities. Second, functional characterization of the GM (e.g., metabolomic profiles and functional gene expression) and immune cell function (e.g., cytokine secretion) was not available, limiting the ability to investigate the underlying molecular mechanisms. Third, the mediation analysis assumed linear relationships and the absence of unmeasured confounding, assumptions that may not fully capture the complexity of biological systems. Finally, IM GWAS did not include detailed clinical information, such as disease severity, age at onset, or complications, which may have influenced the interpretation of the findings.

The identification of *o__Burkholderiales.f__Oxalobacteraceae* and *g__Oxalobacter.s__Oxalobacter_formigenes* as protective taxa suggests that these microorganisms, or strategies aimed at promoting their abundance, warrant further investigation as potential approaches for IM prevention through modulation of the gut microbiota. Likewise, immune biomarkers such as CD62L^+^ pDCs and IgD^−^ CD38dim B cells may help identify individuals at increased risk of IM, including immunocompromised individuals and EBV-seronegative adolescents, although their clinical utility requires prospective validation. The gut-immune axis identified in this study also provided a potential framework for developing novel therapeutic strategies, including interventions that enhance beneficial microbial functions through prebiotics, probiotics, or dietary modification, or that modulate intermediate immune cell activity, such as promoting CM CD8br T-cell activation or optimizing hematopoietic stem cell proliferation.

Future research should validate these findings in non-European cohorts to enhance generalizability, integrate metabolomic and transcriptomic data to elucidate the molecular mechanisms underlying gut microbiota-immune cell-IM interactions, confirm the functional effects and regulatory pathways of the key GM taxa and immune cell traits using germ-free mouse models and cell co-culture experiments, investigate the effects of gut microbiota-immune interactions on IM severity and chronic complications (e.g., chronic fatigue syndrome and lymphoma), and ultimately conduct clinical trials to assess the efficacy of probiotic supplementation or immunomodulatory interventions for IM prevention.

## Conclusion

This two-sample MR study identified causal relationships among GM, immune cell traits, and IM. Three GM taxa (one risk-associated and two protective) and 22 immune cell traits were causally associated with IM. Specifically, the protective effect of *o__Burkholderiales.f__Oxalobacteraceae* was partially mediated by CM CD8br %CD8br and HSC AC. These findings suggest that microbiota-targeted interventions, such as probiotics, may represent a potential strategy for IM prevention. However, these translational implications require validation in experimental and clinical studies and in diverse populations.

## Data Availability

The GWAS summary statistics for infectious mononucleosis, gut microbiota, and immune cell traits used in this study are publicly available from the IEU Open GWAS database and previously published datasets. All analysis scripts are available from the corresponding author upon reasonable request.

## Supplementary Materials

Click to view [pdf].
