# Supplementary material for: Gut microbiota, immune cell traits, and infectious mononucleosis: a two-sample mendelian randomization and mediation analysis
Source: Braz J Med Biol Res. 2026 Nov 9;59:e15657. doi: 10.1590/1414-431X2026e15657 (PMC13747225; doi:10.1590/1414-431X2026e15657)
Supplement: Supplementary file 1 [file 1414-431X-bjmbr-59-e15657-suppl.pdf]

Figure S1. Forest plot of the causal effects of immune cell traits on the risk of infectious mononucleosis (IM) derived from the inverse variance weighted method. nsnp: number of single nucleotide polymorphisms; OR: odds ratio; CI: confidence interval.

exposure	outcome	nsnp	method	pval	OR(95% CI)
IgD− CD38dim %B cell	IM	19	Inverse variance weighted	**0.012**	0.898 (0.825 to 0.977)
HSC AC	IM	15	Inverse variance weighted	**0.046**	0.928 (0.863 to 0.999)
CD33br HLA DR+ CD14dim %CD33br HLA DR+	IM	20	Inverse variance weighted	**0.037**	0.931 (0.870 to 0.996)
CM CD8br %CD8br	IM	29	Inverse variance weighted	**0.020**	0.917 (0.852 to 0.986)
TD CD8br AC	IM	17	Inverse variance weighted	**0.039**	1.112 (1.006 to 1.230)
HLA DR+ CD4+ AC	IM	20	Inverse variance weighted	**0.043**	1.071 (1.002 to 1.145)
Granulocyte %leukocyte	IM	21	Inverse variance weighted	**0.024**	0.904 (0.829 to 0.987)
CD28+ CD45RA+ CD8dim %CD8dim	IM	19	Inverse variance weighted	**0.001**	1.129 (1.050 to 1.215)
CD39+ CD8br %T cell	IM	19	Inverse variance weighted	**0.041**	0.918 (0.846 to 0.996)
CD45RA− CD28− CD8br %CD8br	IM	19	Inverse variance weighted	**<0.001**	1.009 (1.004 to 1.015)
BAFF−R on IgD− CD38br	IM	15	Inverse variance weighted	**0.019**	0.910 (0.841 to 0.985)
CD62L on CD62L+ plasmacytoid DC	IM	13	Inverse variance weighted	**0.001**	1.150 (1.056 to 1.251)
CD3 on HLA DR+ T cell	IM	23	Inverse variance weighted	**0.031**	1.070 (1.006 to 1.138)
CD16−CD56 on HLA DR+ NK	IM	20	Inverse variance weighted	**0.044**	0.921 (0.850 to 0.998)
CD28 on CD39+ secreting Treg	IM	16	Inverse variance weighted	**0.017**	1.104 (1.018 to 1.197)
CD28 on CD45RA− CD4 not Treg	IM	13	Inverse variance weighted	**0.005**	0.903 (0.841 to 0.969)
CD28 on CD28+ CD45RA+ CD8br	IM	13	Inverse variance weighted	**0.017**	0.908 (0.838 to 0.983)
CD127 on granulocyte	IM	30	Inverse variance weighted	**0.032**	1.068 (1.006 to 1.135)
CD25 on CD45RA− CD4 not Treg	IM	19	Inverse variance weighted	**0.027**	1.128 (1.014 to 1.255)
CD25 on CD39+ CD4+	IM	17	Inverse variance weighted	**0.006**	0.930 (0.883 to 0.979)
FSC−A on NKT	IM	18	Inverse variance weighted	**0.049**	0.904 (0.818 to 0.999)
CD4 on activated Treg	IM	19	Inverse variance weighted	**0.035**	1.096 (1.007 to 1.193)

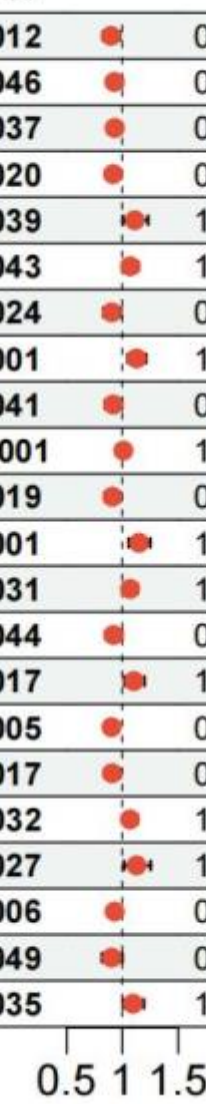

Table S1. Sensitivity analysis between 3 gut microbiota and infectious mononucleosis.

Exposure	Heterogeneity				Pleiotropy		MR-PRESSO
	Method	Cochran's Q	Q-df	P-value	MR-Egger intercept	P-value	P-value
g__Lachnospiraceae_noname.s__Lachnospiraceae_bacterium_8_1_57FAA	MR Egger	12.372	15	0.651	–0.038	0.158	0.572
	IVW	14.584	16	0.555			
o__Burkholderiales.f__Oxalobacteraceae	MR Egger	3.450	7	0.840	0.050	0.443	0.854
	IVW	4.111	8	0.847			
g__Oxalobacter.s__Oxalobacter_formigenes	MR Egger	3.457	7	0.840	0.050	0.444	0.869
	IVW	4.113	8	0.847			

IVW: inverse variance weighted; MR Mendelian randomization; MR-PRESSO: Mendelian randomization pleiotropy residual sum and outlier.

Table S2. Sensitivity analysis between gut microbiota and mediator.

Exposure	Outcome	Heterogeneity				Pleiotropy		MR-PRESSO
		Methods	Cochran's Q	Q-df	P-value	MR-Egger intercept	P-value	P-value
o__Burkholderiales.f__Oxalobacteraceae	CM CD8br %CD8br	MR Egger	12.898	7	0.075	0.015	0.825	0.149
		IVW	12.955	8	0.112			
	HSC AC	MR Egger	6.259	7	0.510	–0.045	0.497	0.613
		IVW	6.772	8	0.561			

MR-PRESSO: Mendelian randomization pleiotropy residual sum and outlier; HSC AC: hematopoietic stem cell absolute count; IVW: inverse variance weighted.

Table S3. Reverse Mendelian randomization (MR): effect of infectious mononucleosis (IM) liability on gut microbiota and immune cells.

Exposure	Outcome	Method	nSNP	β	SE	P-value	OR	OR (95%CI)	
								lower	upper
IM	*o__Burkholderiales.f__Oxalobacteraceae*	MR Egger	14	0.256	0.244	0.315	1.291	0.801	2.081
		Weighted median	14	–0.029	0.086	0.738	0.971	0.820	1.151
		IVW	14	–0.029	0.064	0.647	0.971	0.857	1.100
		Simple mode	14	–0.152	0.158	0.353	0.859	0.631	1.170
		Weighted mode	14	–0.057	0.147	0.706	0.945	0.709	1.260
	HSC AC	MR Egger	27	–0.017	0.079	0.827	0.983	0.842	1.147
		Weighted median	27	–0.053	0.073	0.470	0.949	0.822	1.095
		IVW	27	0.002	0.047	0.964	1.002	0.914	1.098
		Simple mode	27	0.018	0.111	0.871	1.018	0.819	1.266
		Weighted mode	27	–0.038	0.065	0.562	0.963	0.848	1.093
	CM CD8br %CD8br	MR Egger	27	–0.031	0.061	0.619	0.970	0.860	1.094
		Weighted median	27	0.005	0.053	0.922	1.005	0.906	1.116
		IVW	27	0.018	0.036	0.618	1.018	0.948	1.093
		Simple mode	27	0.002	0.073	0.976	1.002	0.868	1.157
		Weighted mode	27	0.010	0.053	0.847	1.010	0.911	1.120

HSC AC: hematopoietic stem cell absolute count. IVW: inverse variance weighted.
